# Endoscopic full-thickness clip closure of a duodenal fistula secondary to foreign body impaction

**DOI:** 10.1055/a-2747-4679

**Published:** 2025-12-08

**Authors:** Kan Chen, Jianwei Zhu, Yilong Wang, Feng Liu

**Affiliations:** 1 278245 Digestive Endoscopy Center, Shanghai Tenth People’s Hospital, Tongji University School of Medicine, Shanghai, China

A 38-year-old man presented with abdominal discomfort after accidentally swallowing three iron wires wrapped with infusion tubing. Although the exact timing was unclear, the patient reported that the ingestion had occurred at least 2 months before. Preoperative abdominal X-ray revealed three approximately 13-cm-long metallic objects in the descending and horizontal portions of the duodenum ( Fig. 1 ). Abdominal computed tomography (CT) showed that one of the wires was likely embedded in the wall of the horizontal duodenum, causing localized inflammatory exudate without evidence of perforation ( Fig. 2 ).

**Fig. 1:**
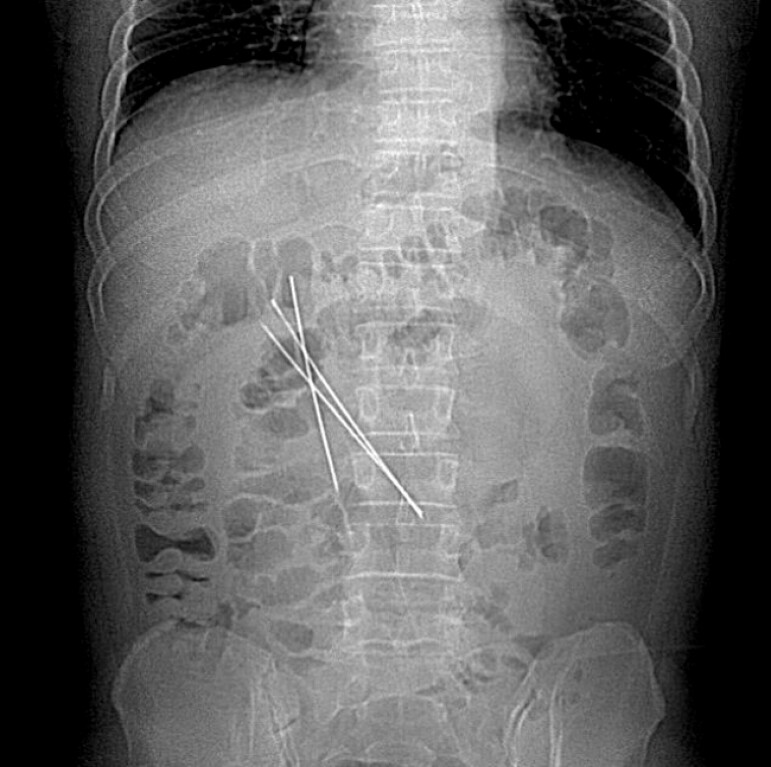
X-ray revealed three metallic objects in the descending and horizontal portions of the duodenum.

**Fig. 2:**
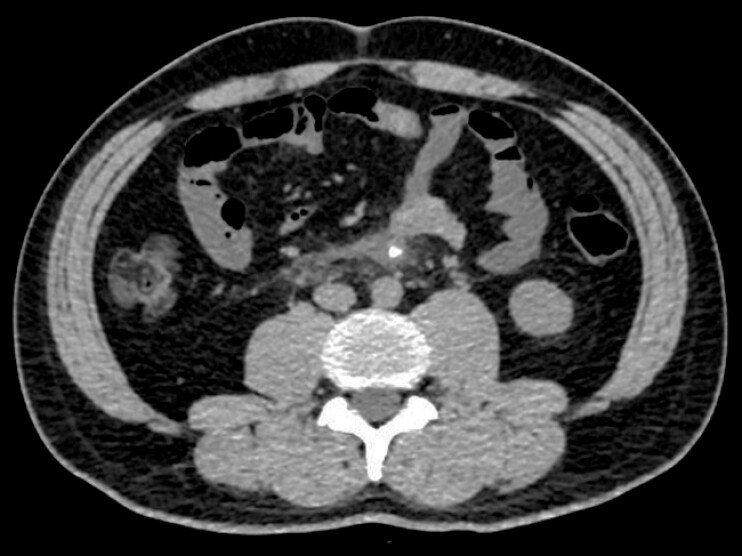
Abdominal CT showed that one of the wires was likely embedded in the wall of the horizontal duodenum, causing localized inflammatory exudate without evidence of perforation. CT, computed tomography.

Endoscopic intervention was performed to remove the foreign bodies. Two freely movable wires were successfully extracted. The third wire was found to be impacted at both ends – in the descending and horizontal duodenum. Bubbling observed at the insertion site in the horizontal portion suggested a microperforation or fistula tract. The oral end of the wire was carefully dislodged and removed ( Fig. 3 ). Subsequent examination revealed a deep ulcerated depression (approximately 3 mm × 8 mm) at the original embedded site in the descending duodenum, with bloody and purulent exudate, surrounded by fibrotic and friable tissue that was unsuitable for conventional clip closure. Given the chronic nature and unfavorable tissue conditions of the defect, the fistula was successfully closed using an over-the-scope clip (OTSC) system, which provides a broader and more secure closure ( Video 1 ).

**Fig. 3:**
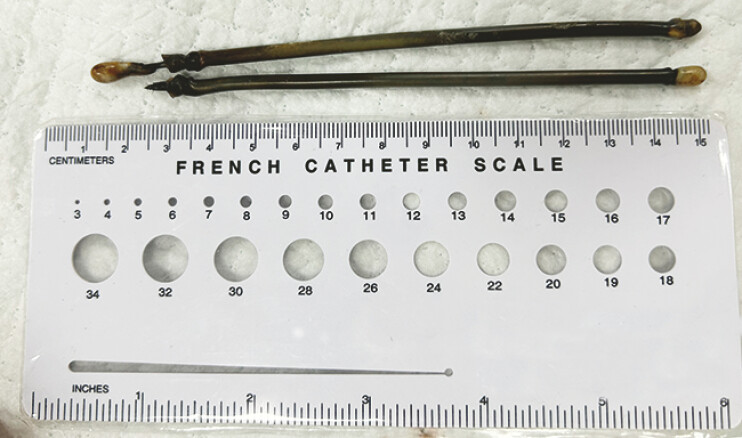
A photograph of the second and third retrieved foreign bodies, with a graduated ruler.

Endoscopic full-thickness clip closure of a duodenal fistula secondary to foreign body impaction.Video 1

Postoperatively, the patient developed mild fever and leukocytosis but reported no significant abdominal pain. Follow-up CT confirmed the absence of free air ( Fig. 4 ). The patient responded well to antibiotic therapy, with resolution of fever and normalization of laboratory parameters, and was discharged on the following day.

**Fig. 4:**
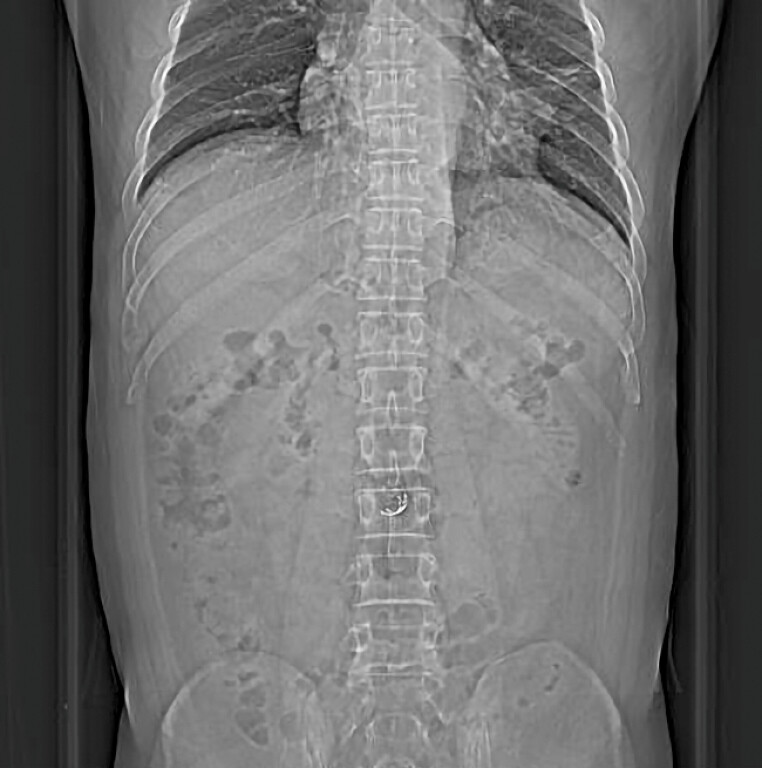
A follow-up abdominal CT scout view showing the absence of free air. CT, computed tomography.

Endoscopy_UCTN_Code_TTT_1AO_2AO

**Correction**

**Correction: Endoscopic full-thickness clip closure of a duodenal fistula secondary to foreign body impaction** Chen Kan, Zhu Jianwei, Wang Yilong et al. Endoscopic full-thickness clip closure of a duodenal fistula secondary to foreign body impaction. Endoscopy 2025; 57: E1413–E1414, doi:10.1055/a-2747-4679 In the above-mentioned article the authorship has been corrected. Correct is that Kan Chen and Jianwei Zhu contribute equally. This was corrected in the online version on December 17, 2025.

## Notes

Endoscopy E-Videos https://eref.thieme.de/e-videos
*E-Videos* is an open access online section of the journal *Endoscopy* , reporting on interesting cases and new techniques in gastroenterological endoscopy. All papers include a high-quality video and are published with a Creative Commons CC-BY license. Endoscopy E-Videos qualify for HINARI discounts and waivers and eligibility is automatically checked during the submission process. We grant 100% waivers to articles whose corresponding authors are based in Group A countries and 50% waivers to those who are based in Group B countries as classified by Research4Life (see: https://www.research4life.org/access/eligibility/ ). This section has its own submission website at https://mc.manuscriptcentral.com/e-videos .

## Notes

Correction **Correction: Endoscopic full-thickness clip closure of a duodenal fistula secondary to foreign body impaction** Chen Kan, Zhu Jianwei, Wang Yilong et al. Endoscopic full-thickness clip closure of a duodenal fistula secondary to foreign body impaction. Endoscopy 2025; 57: E1413–E1414, doi:10.1055/a-2747-4679 In the above-mentioned article the authorship has been corrected. Correct is that Kan Chen and Jianwei Zhu contribute equally. This was corrected in the online version on December 17, 2025.

