# Correction: Endoscopic full-thickness clip closure of a duodenal fistula secondary to foreign body impaction

**DOI:** 10.1055/a-2771-8464

**Published:** 2025-12-18

**Authors:** Kan Chen, Jianwei Zhu, Yilong Wang, Feng Liu

**Affiliations:** 1 278245 Digestive Endoscopy Center, Shanghai Tenth People’s Hospital, Tongji University School of Medicine, Shanghai, China



In the above-mentioned article the authorship has been corrected. Correct is that Kan Chen and Jianwei Zhu contribute equally. This was corrected in the online version on December 17, 2025.

